# Innovative strategies and challenges mosquito-borne disease control amidst climate change

**DOI:** 10.3389/fmicb.2024.1488106

**Published:** 2024-11-05

**Authors:** Yuan Zhang, Minhao Wang, Mingliu Huang, Jinyi Zhao

**Affiliations:** ^1^Ningbo Research Institute of Ecological and Environmental Sciences, Ningbo, China; ^2^Department of Chemistry, University of Liverpool, Liverpool, United Kingdom; ^3^Chou Io Insect Museum, Ningbo Yinzhou Cultural Relics Protection and Management Center, Ningbo, China; ^4^Botnar Research Centre, University of Oxford, Oxford, United Kingdom

**Keywords:** vector, *Aedes aegypti*, *Aedes albopictus*, yellow fever virus, insecticide resistance

## Abstract

The revival of the transmission dynamics of mosquito-borne diseases grants striking challenges to public health intensified by climate change worldwide. This inclusive review article examines multidimensional strategies and challenges linked to climate change and the epidemiology of mosquito-borne diseases such as malaria, dengue, Zika, chikungunya, and yellow fever. It delves into how the biology, pathogenic dynamics, and vector distribution of mosquitoes are influenced by continuously rising temperatures, modified rainfall patterns, and extreme climatic conditions. We also highlighted the high likelihood of malaria in Africa, dengue in Southeast Asia, and blowout of Aedes in North America and Europe. Modern predictive tools and developments in surveillance, including molecular gears, Geographic Information Systems (GIS), and remote sensing have boosted our capacity to predict epidemics. Integrated data management techniques and models based on climatic conditions provide a valuable understanding of public health planning. Based on recent data and expert ideas, the objective of this review is to provide a thoughtful understanding of existing landscape and upcoming directions in the control of mosquito-borne diseases regarding changing climate. This review determines emerging challenges and innovative vector control strategies in the changing climatic conditions to ensure public health.

## Background

Mosquito-borne diseases (MBDs) have long been a substantial burden for global healthcare, causing morbidity and mortality on a large scale (Cella et al., 2019). Different mosquito species, such as *Anopheles*, *Culex*, and *Aedes*, act as vectors carrying disease-causing pathogens and transmit them to the host, including humans and other animals. Approximately 2.5–9.3% of 3,500 mosquito species are related to human disease, with 76% of all known species belonging to the above-mentioned mosquito species (Gizaw et al., 2024). Rising temperatures, changed precipitation patterns, and shifting ecosystems are some of the ways that climate change is affecting mosquito-borne diseases. Higher temperatures can quicken mosquito life cycles, which can hasten the spread of diseases like the Zika virus and malaria. More rainfall increases standing water, which improves breeding sites, especially in urban areas. Furthermore, climate change broadens the geographic range of mosquitoes, enabling them to invade previously unaffected regions, a phenomenon that is becoming more and more evident in places like Europe and North America, where species like *Aedes albopictus* are emerging.

**Figure 1:**
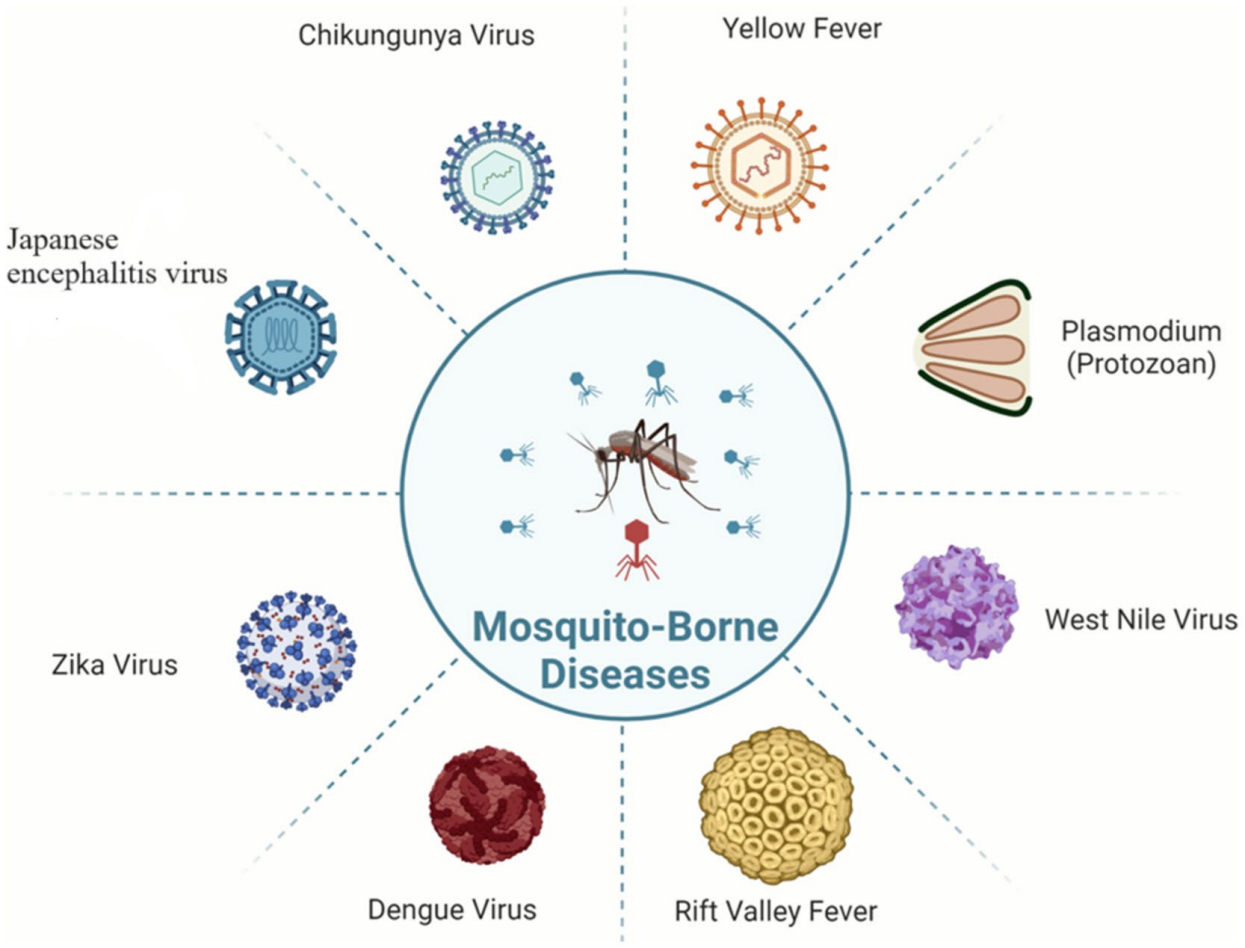
Pathogens of various mosquito-borne diseases.

The control and prevention of MBDs become very difficult due to this vector-borne transmission relationship between vector and host (Figure 1). The number of MBD cases worldwide is very high; in 2020, malaria alone accounted for approximately 241 million illnesses and 627,000 deaths (World Health Organization, 2021).

As greenhouse gases (GHGs) have increased since the preindustrial era, this has led to changes in our climate. It is a worldwide emerging issue and has great impacts on health, the environment, the ecosystem, agriculture, as well as the global economy (Fischer et al., 2020). Anthropogenic greenhouse gas emissions have raised the global mean temperature by about 1°C compared with preindustrial levels. The ugly truths: a 1°C rise has resulted in less cold days and nights, more warm days and nights, increased extreme heat events, and less snow cover—fastest global sea level rate ever recorded (Rosado et al., 2024). While the degree of global warming has been highly diverse, combating a problem as complex and multifaceted in more than just physical terms. The intensity or infectivity of water-related mosquito-borne arboviral diseases (dengue, Zika, and chikungunya) and parasitic infections (malaria), together with the population dynamics of vectors responsible for their transmission, can be directly influenced by climate change-induced changes associated with these settings (Van de Vuurst and Escobar, 2023). Mosquito-borne diseases cause over 1 billion diagnosed cases and more than 1 million deaths worldwide (Table 1) each year (Klingelhofer et al., 2023; Naserrudin et al., 2023).

**Table 1:** Overview of major mosquito-borne diseases worldwide.

Disease	Pathogen	Primary host	Major breakout countries	Citation
Dengue fever	Dengue virus	Humans	Thailand, Philippines, Brazil, India	Gubler (1998)
Zika virus	Zika virus	Humans	Brazil, Colombia, Puerto Rico, Thailand	Kazmi et al. (2020)
Chikungunya fever	Chikungunya virus	Humans	India, Kenya, Sri Lanka, Italy	Powers and Logue (2007)
Yellow fever	Yellow fever virus	Primates, humans	Brazil, Angola, the Democratic Republic of Congo, Uganda	Monath (2007)
West Nile fever	West Nile virus	Birds, humans	United States, Egypt, Israel, India	Molaei and Andreadis (2006)
Malaria	Malaria (*Plasmodium* spp.)	Humans	Nigeria, the Democratic Republic of Congo, India, Mozambique	Monroe et al. (2022)
Japanese encephalitis	Japanese encephalitis virus	Pigs, birds, humans	India, China, Japan, Thailand	Saxena et al. (2013)
Saint louis encephalitis	Saint louis encephalitis virus	Birds, humans	United States, Mexico, Central America	Corrin et al. (2021)
Ross river fever	Ross river virus	Humans	Australia, Papua New Guinea, Solomon Islands	Mackenzie et al. (2017)
Barmah forest fever	Barmah forest virus	Humans	Australia, Papua New Guinea, Africa	Hu (2005)

Intense changes that have already been observed can dramatically amplify if the present trends in greenhouse emissions remain constant and predict an increase of about 4–5°C above preindustrial levels for mean global temperature by the end of this century (Lovey and Schlagenhauf, 2023). The Intergovernmental Panel on Climate Change (IPCC), which is an international body composed of representatives of world’s governments, makes this explicit in its fifth assessment report: emissions need to reach zero. One of the biggest challenges that climate change poses, itself a result of perspiration in vain, is its impact on the emergence and spread of vector-borne disease (Fischer et al., 2013; Piovezan-Borges et al., 2020; Xu et al., 2020).

## Major mosquito-borne diseases

### Malaria

The major killer parasitic disease among vector-borne diseases is malaria, resulting in approximately 620,000 deaths in 2017 only, and the number of people who die from this deadly mosquito-borne disease annually is more than 400,000 (Samarasekera, 2023). Malaria is a widespread mosquito-borne disease (Table 2) produced by plasmodium parasites and transmitted by Anopheles mosquitoes specifically in sub-Saharan Africa, as 90% of the total mortality and 85% of the total malarial cases mainly occur there (Trujillano et al., 2023). Because of complex life cycle of parasites completing in humans and a mosquito vector involved in both asexual and sexual reproduction mechanisms, it poses a challenge to develop vaccines and drugs for treatment. Despite advances in techniques and strategies such as artemisinin-based combination therapies (ACTs) and insecticide-treated nets (ITNs), malaria still remains a major threat to public health globally, causing a huge number of cases and deaths annually (Carlson et al., 2023).

**Table 2:** Malaria incidence and mortality in different countries (2000–2023).

Year	Country	Estimated malaria cases (millions)	Estimated malaria deaths (thousands)	References
2000	Nigeria	35	170	WHO World Malaria Report 2005
2005	Democratic Republic of Congo (DRC)	20	105	WHO World Malaria Report 2006
2010	India	15	20	WHO World Malaria Report 2011
2015	Uganda	14	85	WHO World Malaria Report 2016
2018	Tanzania	12	59	WHO World Malaria Report 2019
2020	Mozambique	10	42	WHO World Malaria Report 2021
2022	Ghana	9	30	WHO World Malaria Report 2023
2023	Burkina Faso	8.5	28	WHO World Malaria Report 2023

### Dengue fever

Dengue is the second most febrile infection after malaria among mosquito-borne diseases (Table 3), as the rate of global incidence of dengue enhanced exponentially over recent decades (Andriamifidy et al., 2019; Cella et al., 2019; Filali and D'Acremont, 2023). The causative agent of this disease is dengue virus (DENV), including four different types of serotypes (Agha et al., 2017). DENV enters the human primarily through *Aedes aegypti* and *Aedes albopictus* mosquitoes, particularly in rainy season. Dengue is considered to be the most predominant and deadly disease, as over 2.5 billion individuals are susceptible to dengue every year, continuing to prevail in several countries (Bal and Sodoudi, 2020; Malavige et al., 2023).

**Table 3:** Establishment and mortality of dengue fever (2000–2023).

Year	Country	Deaths (approx.)	References
2000	United States	1	Johnson et al. (2014)
2001	India	100	Gubler (2001)
2002	Brazil	500	Bezerra and Sorpreso (2016)
2003	Thailand	200	Nguyen et al. (2003)
2004	Malaysia	300	Lum et al. (2005)
2005	Philippines	400	Bombieri and Gubler (2006)
2006	Vietnam	600	Nguyen et al. (2007)
2007	Indonesia	700	Wahyuni et al. (2011)
2008	Sri Lanka	150	Weerakoon et al. (2008)
2009	Nepal	30	Maharjan et al. (2009)
2010	Pakistan	120	Nisar et al. (2011)
2011	Bangladesh	150	Hossain et al. (2012)
2012	Saudi Arabia	20	Khan et al. (2013)
2013	South Africa	40	Getts et al. (2014)
2014	French Polynesia	10	Inzlicht et al. (2015)
2015	Réunion	25	Nicolas et al. (2017)
2016	Mauritius	15	Muli et al. (2018)
2017	Seychelles	5	Gibson et al. (2018)
2018	Australia	3	Li et al. (2019)
2019	Fiji	8	Aporosa et al. (2020)
2020	Cambodia	12	Pegu et al. (2021)
2021	Laos	10	Vong et al. (2022)
2022	Timor-Leste	7	Blomhoff et al. (2023)
2023	French Guiana	6	Martin et al. (2023)

### Zika virus

The Zika virus, which is likewise spread by *Aedes* mosquitoes, obtained the growing attention of the people of scientific community during Brazil and America’s outbreaks in 2015–2016 before it was considered an illness restricted to limited geographical areas (Accoti et al., 2023; Shariff et al., 2023). Zika infections can result in neurological problems like Guillain-Barré syndrome and serious birth defects like microcephaly, despite the fact that they frequently show no symptoms at all or only moderate ones. Zika’s quick spread brought to light the necessity of effective surveillance and reaction systems for newly developing mosquito-borne dangers (Blagrove et al., 2020; Ngonghala et al., 2021). The fact that Zika has returned to areas that were previously virus-free highlights how dynamically vector-borne disease transmission occurs in a changing climate (Cortes et al., 2023).

### Chikungunya

Chikungunya is an alpha virus carried by Aedes mosquitoes. Symptoms include excruciating joint pain, fever, and rashes. After being isolated for the first time, CHIKV seldom caused outbreaks in Africa and Asia for the next 50 years (Delrieu et al., 2023; Simon et al., 2023). Despite having low death rates, CHIKV causes severe morbidity, which has a major negative influence on the quality of life of those who are infected and causes large financial losses, particularly in developing nations (Krokovsky et al., 2022). The devastating nature of the disease and the absence of precise antiviral treatments emphasize the need to take steps to control and benefit public health (Nosrat et al., 2021).

### Yellow fever

Yellow fever, produced by the yellow fever virus (YFV) and carried by an arbovirus of the family *Flaviviridae*, genus *Flavivirus*, is a prominent hazard in Africa and South America (Agha et al., 2017). Due to the lack of proper vaccination and sustained immunity in population, outbreaks of yellow fever occur subsequently (Ewing et al., 2021). Yellow fever has clinical manifestations characterized by fever, muscle pain, headache, nausea, vomiting, jaundice, and fatigue. The possibility of death for yellow fever-infected people is estimated to be 47%, as such a variety of clinical symptoms makes its diagnosis difficult (Sadeghieh et al., 2021; Gianchecchi et al., 2022).

## Impact of climate change on mosquito-borne disease dynamics

Climate can affect the dynamics, geographic distribution, and comeback of vector-borne diseases in a number of ways, including direct effects on pathogens, vectors, non-human hosts, and humans. In addition to having a direct impact on specific species, climate change drastically alters ecological habitats, particularly in urban areas where non-human hosts and vectors may thrive or fail (Carreto et al., 2022; Mojahed et al., 2022; Oberlin and Wylie, 2023).

### Climate change factors

**Figure 2:**
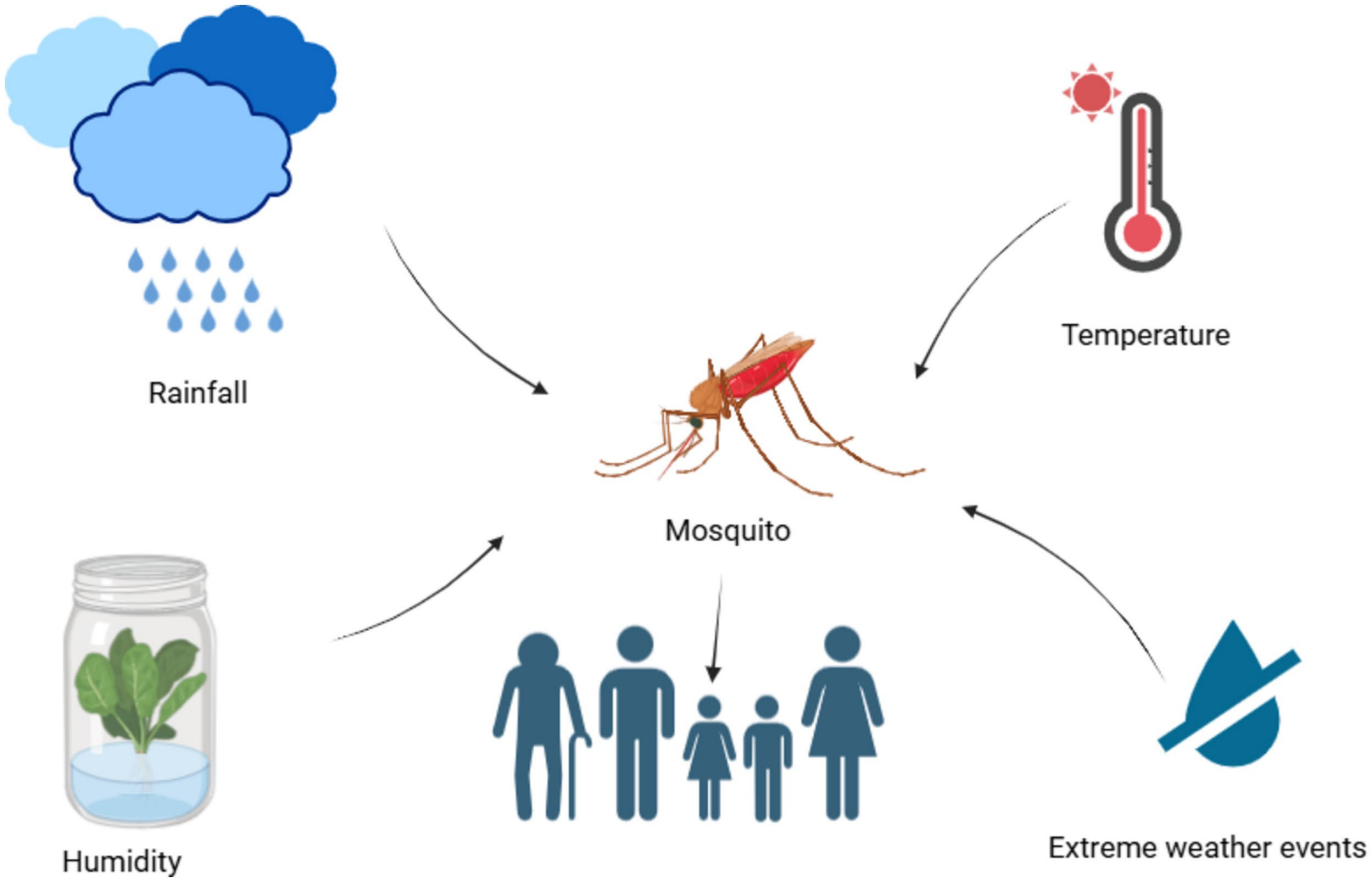
Climatic factors affecting the mosquito-borne diseases in humans.

Tenacity or appearance of a vector-borne disease is dependent on a suitable climate. Non-climatic factors, along with the climatic factors (Figure 2), are also responsible for thrive of vector-borne diseases in a particular geographic region (Edillo et al., 2024).

### Rising temperatures

**Figure 3:**
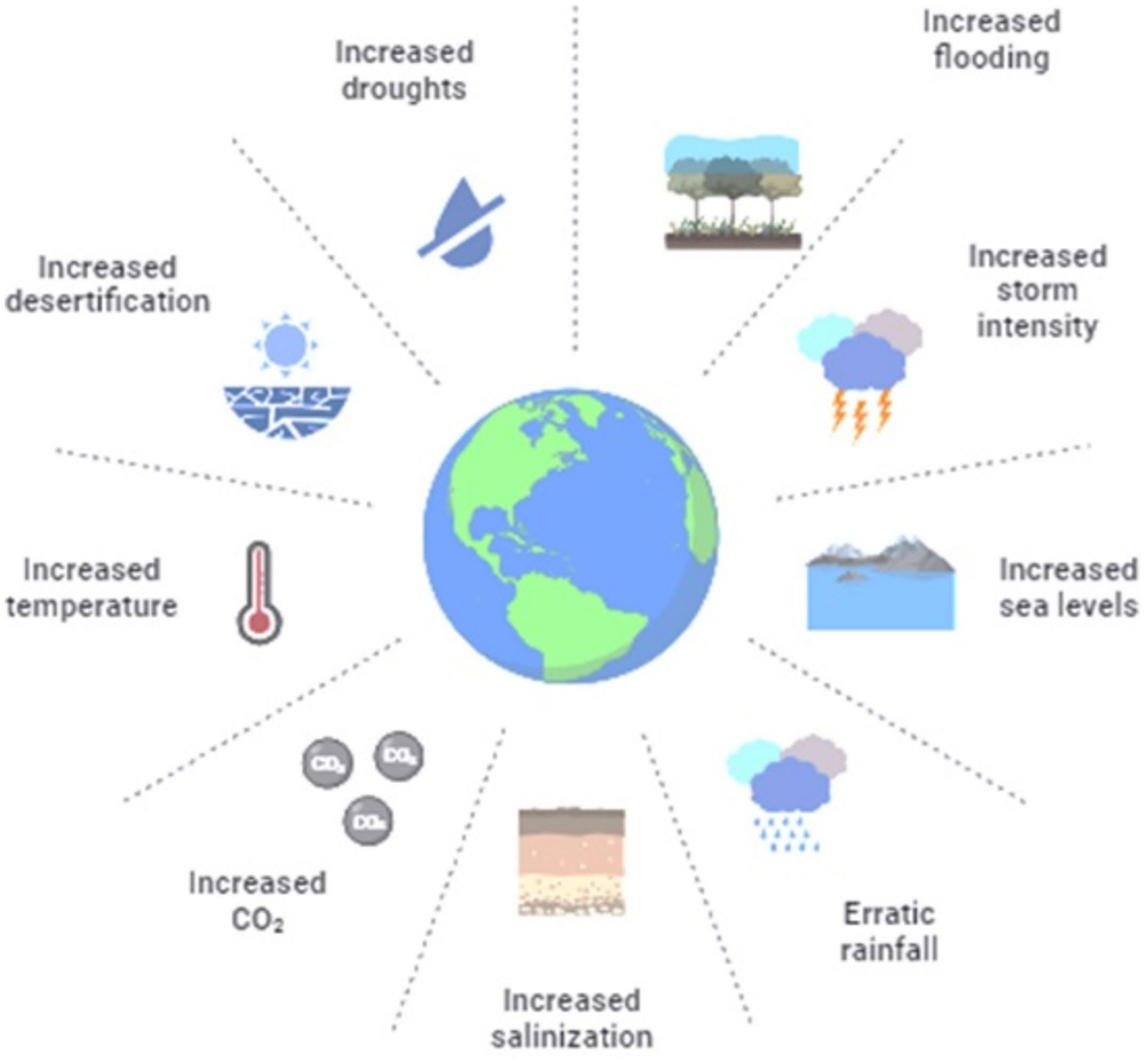
Environmental factors involved in global climate change.

Malaria and other diseases carried by mosquitoes are enormously subtle to environmental factors (Figure 3). An important factor influencing the life cycle characteristics of mosquitoes is temperature, along with humidity, fecundity, and biting frequency (Gizaw et al., 2024). The chance of human–mosquito transmission and the pace at which disease develops inside mosquitoes (the extrinsic incubation period) are similarly influenced by temperature (Reinhold et al., 2018; Ciota and Keyel, 2019; Ziegler et al., 2023). Consider the laboratory-based performance of dengue vector *Aedes aegypti*, for instance, the life-history traits expressing the temperature impact curve. These include the survival rate of egg and adult phase is near linear, the first ranging from 0% at 15°C and reaching 90% at 20°C before declining to 60% at 35°C. The second trait consists of the time of egg to adult phase development ranging from over 60 days at 15°C to 12 days at 20°C, continuous and a slow decline to 6 days at 27–34°C.

The third trait is the percentage of mosquitoes that complete a blood meal in the first 30 min of the host being available, rapidly increasing to a peak of about 50% between 22°C and 28°C, then declining, and reaching almost 0% at 33°C (Ryan et al., 2021; Lyberger et al., 2023).

As the planet warms, the frequency and transmission rate of mosquito-borne diseases will extend in some endemic areas because of the blowout of mosquitoes and viruses to higher altitudes and latitudes. The widespread expansion of dengue vector has already been revealed into more temperate regions. The probability of decline in mosquito-borne diseases in endemic areas is also possible with the extreme rise in temperature, with vector survival and feeding habitat in danger (Rahmani et al., 2022; Ismail et al., 2023).

### Altered precipitation

There is a complex bond between precipitation and the occurrence of mosquito-borne diseases (Chowell et al., 2019). Breeding sites of mosquitoes will flourish with increased rainfall; however, the drought conditions seem to be favorable for many mosquito species population because of increased water storage, which provides more aquatic breeding sites to promote vector abundance. Diseases spread by mosquitoes are strongly impacted by altered precipitation patterns. For instance, more rainfall can result in more mosquito-breeding grounds, as demonstrated by the dengue fever epidemic in Southeast Asia, where longer monsoon seasons boost the disease’s transmission rates. On the other hand, a drought may drive mosquitoes to congregate nearer sources of water, which could raise the danger of illnesses like the West Nile virus in the United States. For public health policies to be effective, it is imperative to comprehend these dynamics (Coalson et al., 2021; Lowe et al., 2021).

### Extreme weather events

Although the role of extreme climate events such as floods, drought, cold, and heat waves in the mosquito-borne disease transmission is not well defined, it remains a matter of concern for scientific communities as well (Coalson et al., 2021; Magallanes et al., 2024). Previous studies revealed that the breeding habitats increased with the increased rainfall, but extreme rainfall and floods are not favorable for the abundance of mosquito vectors as it may flush all the breeding sites to overwhelm many populations of different mosquito species. For instance, it was observed that extreme rainfall removed all the breeding sites for *Aedes aegypti* in Singapore during 2014–2015, diminishing the risk of dengue fever outbreak 6 weeks following rainfall (Baril et al., 2023; Van de Vuurst and Escobar, 2023). There is a correlation between ecosystems and extreme climatic events, as they may improve or degrade the vector habitats and the competition, which are directly linked with prompted vector pathogens or vector predators (Edillo et al., 2024).

### Regional impacts of climate change on mosquito-borne diseases

A range of regional and local signals indicate that climate change has either already affected or will affect the distribution, spread, and incidence of vector-borne diseases. For instance, models based on time-series analysis of monthly malaria case data in the highlands of Colombia and Ethiopia suggest a northward and upward spread along elevational lines, with higher altitudes experiencing warmer climate years observed as future trends regarding increased numbers of anemia cases at parasitemias >100 per 1 L. Travel, trade, or migration can spread pathogens into non-endemic areas (Cazelles et al., 2023).

### Resurgence of malaria in high-altitude Africa

Africa is home to a large burden of mosquito-acquired infections, especially malaria. Global warming and climate change have increased the movement of malaria into highland areas where cold temperatures used to less prevail less often (Tchouassi et al., 2022; Matute and Cooper, 2023). Increased malaria transmission has been noted in locations where high temperatures are suitable for Anopheles mosquitoes. Not only malaria, the continent has to deal with other mosquito-based diseases like dengue and chikungunya (Buchwald et al., 2020). Urbanization and peri-urbanization have been associated with changes in precipitation patterns, leading to the expansion of Aedes colonization and threats, including dengue replacement.

### Dengue incidence in Southeast Asia

In Asia, mosquito-borne diseases represent biting mosquitoes, with dengue as one of its major public health burdens. Rising temperatures and altered rainfall patterns have also been associated with dengue incidence increases in Southeast Asia, as they create conditions for the proliferation of Aedes mosquito-breeding environments. Furthermore, urbanization and land use changes have intensified exposure to mosquito-borne diseases in Asia. The increased urbanization has resulted in an increase in mosquito-breeding sites, such as water storage containers and construction sites, which have facilitated the dissemination of dengue virus along natural arboviral parameters.

### Spread of *Aedes* mosquitoes into temperate regions (Europe, North America)

Mosquito-borne outbreaks, including Zika and dengue, have affected Central and South America. Climate change has affected the dissemination and density of *Aedes* mosquitoes, which is why these diseases are emerging or re-emerging in areas where they had not been reported before (Wint et al., 2022). The El Niño-Southern Oscillation (ENSO) phenomenon, which refers to periodic warm sea surface temperatures in the Pacific Ocean region, has been associated with a rise in mosquito-borne disease cases seen across the Americas. El Niño can impact the distribution of rainfall and the temperature; hence, it may lead to conditions conducive to mosquito breeding if a temporal association occurs with vector-borne disease transmission (Huang et al., 2019).

In Europe, *Aedes albopictus* has been facilitated by rising temperatures and international travel, thereby compromising containment strategies. The mosquito *Aedes albopictus* is a potential vector for dengue, chikungunya, and Zika viruses. These diseases have, in turn, resulted in numerous outbreaks of endemic disease reported within Europe and further underscored the need for sustained surveillance measures to suppress amplified dissemination (Rossati et al., 2015; Lovey and Schlagenhauf, 2023).

## Innovative strategies for mosquito-borne disease control

### Genetic approaches

#### Wolbachia-infected mosquitoes

Naturally occurring endosymbiotic bacteria called Wolbachia exert a significant pressure to reduce the incidence of mosquito-borne diseases by lowering vector competence and delaying the reproduction of viruses like DENV, ZIKV, CHIKV, and YFV (Mushtaq et al., 2024). They are found in a few significant mosquito disease vectors, including *Ae. albopictus*, *Cx. quinquefasciatus*, and anopheline species, which include vectors of malaria such as An. gambiae and *An. coluzzii* but never *A. aegypti*. A *Wolbachia*-infected male mates with an uninfected female, but not the other way around (Minwuyelet et al., 2023). This maternally transmitted bacterium that permits the invasion of host populations can also cause parthenogenesis, or reproduction without males; feminization of males (turning genetic males into females) and cytoplasmic incompatibility, which results in the generation of inviable offspring. *Wolbachia*-infected strains have been successfully applied in many countries including, Brazil, Vietnam, Indonesia, Australia, and Colombia, until now (Mercant Osuna et al., 2023; Hyder et al., 2024). In nations where malaria and arbovirus epidemics are rife, Wolbachia-based management is a potentially effective tactic for managing mosquitoes and the diseases they transmit. It must be carefully evaluated and integrated with biological control initiatives (Turner et al., 2023).

### Genetically modified mosquitoes (GMMs)

Scientists have been working to engineer modified mosquitoes since this century with the aim of reducing disease transmission by mosquitoes. In this respect, some risks are to the human health, and others may affect non-human species (some birds could be at risk due to self-ingestion of contaminated grain by exposed insects) (Ketkar et al., 2019). Today, *Aedes aegypti* are resistant to DDT and have developed resistance over the years for other artificial chemicals made in labs. The most prominent two areas are (1) genetically engineered male mosquitoes so that they do not produce viable offspring, creating a disease-resistant or non-transmissive strain of male and/or female mosquito species, and (2) modifying male and female mosquitoes so that they resist certain diseases or are incapable of transmitting them (do Nascimento Neto et al., 2020; Hoermann et al., 2022). Engineered mosquitoes have also been tested in the field, but this approach was conducted by a biotechnology company (Oxitec) and only phase 1 tests were reported; another proposed gene drive has remained stalled at laboratory scale due to biosafety concerns from even more potential unintended consequences as RNAi targets multiple locations of genome simultaneously. The GM *Anopheles aegypti* 1 male mosquitoes from Oxitec have been genetically altered to kill their larvae unless tetracycline, an antibiotic, is applied to the larvae. GM males mate with wild females, but the offspring are non-viable. The use of genetically modified mosquitoes (GMMs) represents a novel development in vector control. Manipulation of GMMs to prolong mosquito lifespan, induce sterility, or enhance resistance against pathogen infection is feasible (Lu et al., 2023; Hancock et al., 2024).

### Sterile insect technique (SIT)

One method, the sterile insect technique (SIT), releases sterilized male mosquitoes directly into the wild, which reduces mosquito populations by interfering with reproduction. These gene drive technologies could be used to enable GMMs that spread improvements like pathogen resistance genes throughout populations of mosquitoes in order to mitigate certain problems inherent even with biological controls. However, the ecological and ethical impacts must be carefully weighed during GMM deployments (Jimenez-Alejo et al., 2022; Fola et al., 2024).

### Biological control

#### Larvivorous fish and predatory insects

Before the use of DDT, the preferred choice was fish for control of aquatic stages of mosquitoes. Following initial introduction into any available mosquito-breeding habitats, the use of these fish was terminated after inclusion of DDT and subsequently resurrected following resistance to them developed (Enahoro et al., 2020). Indigenous larvivorous fish have been used, but only a few (mostly *Gambusia affinnis* and *Poecilia reticulata*), although several failures were reported in the literature, e.g., rainy seasons increase proportions and enhance other aquatic predators to act as biological control agents of mosquito populations, but synergistic interactions among these natural predators may decrease the diversity (Hustedt et al., 2021). The non-biting adult *Toxorhynchites* is a genus of naturally occurring eavesdroppers, as its larval stage can be very predaceous, making these ideal candidates for potential biological control alternatives to chemical insecticides (Fontoura et al., 2021). Significant steps were taken in their production as biological agents and showed high effectiveness against various mosquito species such as *Ae*. *aegypti*, *Ae*. *albopictus*, and *Cx*. *quinquefasciatus*. It has shown to be operationally feasible in some settings, but there are still many issues that limit its use, i.e., early instar cannibalism, temperature (limited by lower temperatures), and lack of larval habitat overlap between the prey mosquito and predator (Jourdan et al., 2021).

### Protozoan control

*Chilodonella uncinata* is a protozoan parasite that has acquired several good properties of pathogen and microbe. Low to extremely high (25–100%) phototoxic mortality in mosquito larvae. It manifests as highly virulent, desiccation-resistant, and *in vitro* resembling cultures of high reproductive potential. *C. uncinata* has the capability of natural circulation by trans-ovarian transmission, using its mosquito host (Hegazy et al., 2022; Shahbodaghi and Rathjen, 2022).

### Chemical control

#### Long-lasting insecticide-treated nets

Fertilizer-treated fabric, usually pyrethroids, is used in factories to create long-lasting insecticide-treated nets, or LLINs. Based on the drawbacks of conventional insecticide-treated nets (ITNs) and unique fabric technologies, LLINs were created to withstand up to 20 washings while being used in field settings. These LLINs not only act as physical barriers to keep out would-be mosquitoes, but they also repel or kill insects that come into contact with the chemicals coated on the net fabric. As a result, LLINs offer both community-wide and individual-level protection against mosquito-borne illnesses that lasts longer. Long-lasting insecticide-treated nets are seen to be among the best methods for controlling mosquito populations, especially when it comes to preventing malaria (Hancock et al., 2024). The effectiveness of LLINs against the target organism determines which molecules are used, and appropriate application may boost their effectiveness (Enahoro et al., 2020).

Malaria morbidity and death have considerably decreased (by about 50%) in sub-Saharan Africa, where more than 427 million insecticide-treated nets were provided between 2012 and 2014. Over the past 15 years, long-lasting insecticide-treated nets have also contributed to a decrease in malaria cases in pregnant women and children worldwide (Kumala et al., 2022). In a study carried out in the southeast of Iran, groups of LLIN users had a significantly lower prevalence of malaria (up to 97%) than groups of LLIN non-users. However, a number of environmental factors affect how well LLINs prevent diseases spread by mosquitoes. The success of LLINs is also significantly influenced by their accessibility (Feio-Dos-Santos et al., 2024).

### Indoor residual spraying

**Figure 4:**
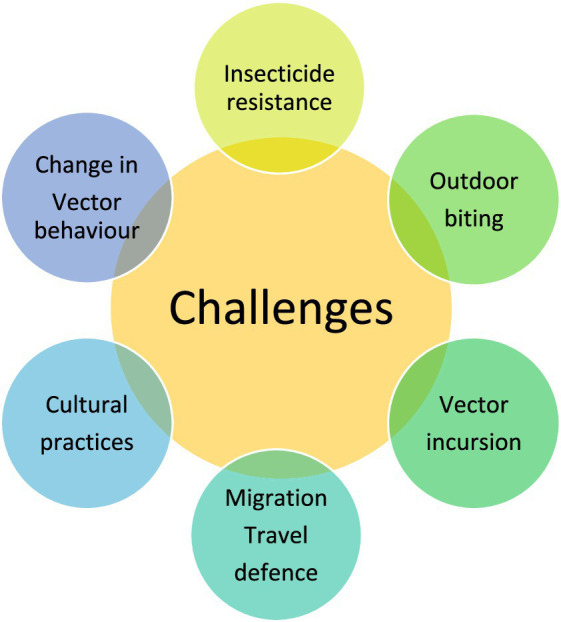
Key challenges in vector control for mosquito-borne diseases.

Indoor residual spraying (IRS) consists of the application of insecticide to surfaces within houses that serve as resting places for mosquitoes. *A. aegypti*, which is predominantly endophilic and anthropophagic. The fact that the procedures require specific training and most are time-consuming. Another limitation is that some methods can be very tedious. Indoor Residual Spraying However, it does not prevent persons bitten by mosquitoes (Weng et al., 2024). Exploiting indoor residual spraying of insecticides alone or in combination with larviciding can lower the mosquito burden and disease incidence (Dulacha et al., 2022). In the Mediterranean region, three recent studies have provided a strong and unequivocal evidence that supports not only IRS as an important component of malaria control strategies in this area but also challenges (Figure 4), the WHO 2006 affirmation, which recommended against its use for protection at higher altitudes anywhere it is still endemic-based on reassuring little or no benefit was associated with consideration of limited areas where vectors are known to be primarily endophytic (Hernandez et al., 2022). Thus, although IRS-based malaria eradication programs substantially reduced the transmission of PM, many more cases remained asymptomatic after the introduction of PCR. As mosquitoes are becoming resistant to pyrethroids, new formulations, such as bendiocarb, have been deployed for the IRS in order to stop vectors from developing insecticide resistance.

### Peridomestic space spraying

It is most widely used around rural housing and involves the pulverization of dichloro diphenyl trichloroethane (DDT) usually throughout homes. By releasing a fine mist of chemicals in the form of insecticides into space, this system kills exclusively adult mosquitoes with no impact at all on immature stages (e.g., eggs or larvae). This technique is mainly used in emergency situations where there are high numbers of adult mosquitoes (Dulacha et al., 2022). One of the most regularly utilized strategies in PDS, though, is an ultra-low volume spray framing a standalone or vehicle-mounted machine. Insecticidal concentrations are typically in the range of 2% (pyrethroids) to 95% (organophosphates), depending on active component content within the formulation. The concentration of these active ingredients varies as a function of the toxicity to the target species. While pyrethrin aerial sprays have a significant impact on non-targets, they do not appear to make an important contribution to water quality issues (Diouf et al., 2022). Mosquitoes can also be infected by Dengue and Chikungunya viruses through trans-ovarian transmission, so residuals available to kill mosquitoes emerging after treatments are potentially interesting from the point of view of these diseases. Consequently, there is a liability to subsequent Dengue and Chikungunya vector control that form the breeding efforts as recurrent treatment (Yin et al., 2021).

### Mosquito repellents

Mosquito repellents are not lethal to mosquitoes, and they only prevent them from biting people. The best known of these are the repellents which virtually eliminate any human scent and thus make them probably efficacious insecticides (Munugoda et al., 2023).

DEET (N, N-diethyl-m-toluamide or N, N-diethyl-3-methylbenzamide) and IR3535 [3-(N-butyl-N-acetyl)-aminopropionic acid] treated clothing among synthetic insect repellents would serve as long-lasting insect repellents. The conventional repellents, due to their synthetic nature, have attracted many criticisms as they promote the development of resistance in mosquitoes for insecticides and have effects on non-targeted organisms, leading to ecosystem imbalance (Contreras-Perera et al., 2021). Therefore, mosquito control measures containing natural repellents derived from plants or fungi/bacteria are recommended for the effective suppression of mosquitoes and to ensure human/environmental safety where local endowment resistance resists them, as well as with further migrations reaching eastern/central Europe. In addition, nanoparticles were used for cotton clothes to act as mosquito repellent, and nanoparticles have shown effectiveness against mosquitoes. They therefore have the potential for use in clean technologies to control malaria-transmitting mosquitoes (Dilani et al., 2022).

### The challenges and need for new vector control

#### Insecticide resistance

The evolution of resistance in mosquitoes to the current insecticides available is one key driver for new tools. The two principal malaria vectors, *Anopheles culicifacies* and *An. stephensi*, have been reported to exhibit different levels of resistance against the three insecticide classes, such as DDT, malathion, and synthetic pyrethroids (Hernandez et al., 2022).

In 52 study districts of nine states from where *An. culicifacies* resistance to various public health insecticides, including DDT (Results data: insecticide susceptibility studies on malaria vectors data). The species culicifacies was first reported as anti-malarial but confirmed resistant to malathion and synthetic pyrethroids except one case where possible resistance is reported (Diouf et al., 2022; Mawejje et al., 2023).

*Anopheles stephensi* and *A. culicifacies* populations from 52 study districts spread across nine states showed resistance in levels that cause potential control failures according to the Centers for Disease Control Light Microscopy Assay while earlier detecting, but when confirmed by LC50 mortality bioassays based on WHO guidelines elsewhere, the situation was worse than many suspected as most mosquitoes were DDT resistant—also evidenced meaning, Evolution of Resistance (EoResistance). More than 80% of the 14 areas under investigation reported having populations that were resistant, or potentially resistant, to synthetic pyrethroids and malathion. Direct observation, binary evaluations, or oral/mortal testing provided evidence for this.

In another study, primary malaria vectors such as *A. stephensi* and *A. fluviatilis* have been reported to be resistant to DDT in eight states of India (Hafsia et al., 2022).

Chikungunya and dengue vectors (*Aedes aegypti* e *Ae. albopictus*) showed resistance to DDT but were highly susceptible to synthetic pyrethroids. The vector of Kala-azar, *Phlebotomus argentipes*, has been reported as DDT-resistant too (Gomez et al., 2022).

### Outdoor biting of vector

The main malaria vectors in India are endophagic, whereas *An. foresti* has been reported to bite outdoors. The most probable suggested reasons are probably due to prolonged use of chemical insecticides both by ITNs and IRS. In fact, tools to control these vectors that bite outdoors are virtually nonexistent for public health programs. It is also important to note that outdoor biting monitoring has not been satisfactory, and therefore the magnitude of this remains unknown (Elmardi et al., 2021).

### Change in vector behavior

Behavioral shifts in malaria-transmitting mosquitoes threaten the efficacy of currently available interventions, including IRS and LLIN, which could reverse recent gains made against malaria transmission (Contreras-Perera et al., 2021). Shift in resting behavior of *An. fluviatilis* occurs exclusively in pentoses mixed with higher degree of anthropogenic (Hinne et al., 2021). Similarly in Madhya Pradesh, outdoor resting behavior of *An. culicifacies* relative to previous years demonstrates a significant shift in resting behavior. These shifts in resting behavior are of considerable importance, as none of the current vector control tools consider exophilic vectors. As a result, the lack of proper tools specifically suited for getting mosquitoes that rest in additional habitats such as cattle sheds or outdoors can exacerbate this problem.

### Cultural practices

Jhum cultivation, or slash-and-burn, is practiced by a sizable portion of the population in the north-east states of India. Lu et al. (2022) reported that Jhum cultivators were at risk of malaria infection compared to non-Jhum cultivators. Jhumias are mosquito-genic and conducive areas for malaria transmission, so their encroachment into virgin forest regions through shifting agriculture makes them vulnerable to contracting the disease (Proestos et al., 2015; Bal and Sodoudi, 2020).

### Newer vector or vector incursion

These days, exotic mosquito vectors are expanding their range and entering new zones. Originally from India, *An. stephensi* is a significant urban malaria vector that has spread to Sri Lanka, posing a danger to the nation’s 2016 malaria-free status. Recent reports on the geographic distribution of *An. stephensi* have come from several locations in Africa, including Djibouti, Ethiopia, Sudan, Somalia, and Nigeria (Tsheten et al., 2021). *Ae. albopictus* and *Ae. aegypti*, which are native to Africa, have spread farther and established themselves in areas of Oceania and Asia, as well as southern Europe and North America, despite considerable efforts by the WHO to limit *An. stephensi* in these regions (Dusfour and Chaney, 2022).

### Migration, travel, and defense area

Malaria vectors could infect non-immune migrants, travelers, and armed forces personnel when they travel or are posted in malaria-endemic areas. This has implications for human migration, where exposure of the population to malaria increases in some parts of India. Mobility was further reported to be an important factor for kala-azar epidemics in Africa and as a key contributor to the epidemic of visceral leishmaniasis (Kala-Azar) in India (Citron et al., 2021). The movement of troops in and out from endemic to holoendemic areas and vice versa; likewise, the spread of new malaria parasite strains, with local epidemics. This is further exacerbated by the forest movement of Army personnel who are not able to directly protect themselves from mosquitoes (Xu et al., 2021). Defense personnel posted in interstate or international border areas are also noted with greater proportion of severe morbidity, attributable to high risks of infection acquisition at these places. It is critical for the strategy and tool to cater to mobile populations like travelers, migrants, defense personnel, etc. (Merakou et al., 2023).

## Conclusion

The nexus of climate change and mosquito-borne diseases presents multifaceted difficulty and requires multifaceted strategy and alternative solutions. The combination of biological data and modeling is thus pushing back the horizons for approaches to mitigate against climate change with impacts on disease transmission, especially in entomology by surveillance (tele-epidemiological attitudes), predictive models, and vector-controlled techniques. However, the formidable challenge of mosquito-borne diseases also underscores how scientific knowledge must evolve with them in a world where nature is changing and movement across borders is increasingly commonplace. By considering climate change adaptation within public health strategies and involving our communities, together we may be better able to safeguard ourselves against mosquito-borne diseases and begin the long journey of understanding what it might take for the Canadian Public Health system to need in a future that is not just uncertain but likely very different.
